# Structure-based neutralizing mechanisms for SARS-CoV-2 antibodies

**DOI:** 10.1080/22221751.2022.2125348

**Published:** 2022-10-09

**Authors:** Qingrui Huang, Xiaonan Han, Jinghua Yan

**Affiliations:** a CAS Key Laboratory of Pathogenic Microbiology and Immunology, Institute of Microbiology, Chinese Academy of Sciences, Beijing, People’s Republic of China; b University of Chinese Academy of Sciences, Beijing, People’s Republic of China

**Keywords:** SARS-CoV-2, neutralizing antibody, neutralizing mechanism, pan-coronavirus vaccine, antibody structure

## Abstract

The devastating economic and public health consequences caused by the COVID-19 pandemic have prompted outstanding efforts from the scientific community and pharmaceutical companies to develop antibody-based therapeutics against SARS-CoV-2. Those efforts are encouraging and fruitful. An unprecedentedly large number of monoclonal antibodies (mAbs) targeting a large spectrum of epitopes on the spike protein has been developed in the last two years. The development of structural biology, especially the cryo-EM technology, provides structural insights into the molecular neutralizing mechanisms of those mAbs. Moreover, neutralizing antibodies are essential in protecting host from infection. Therefore, understanding the antibody neutralizing mechanism is critical for optimizing effective antibody-based therapeutics and developing next-generation pan-coronavirus vaccines. This review summarizes the latest understanding of antibody neutralizing mechanisms against SARS-CoV-2 at the molecular and structural levels.

## Introduction

Since COVID-19 emergence, various SARS-CoV-2 vaccines have been developed at an unprecedented speed, and several have been approved for the general public or emergence use [1-8]. It is demonstrated that neutralizing antibody levels in vaccine recipients positively correlates with vaccine protection efficacy against symptomatic COVID-19 [9]. In the meantime, many neutralizing antibodies have been isolated and developed as therapeutic drugs [8,10-17]. Those antibodies with well-characterized binding modes exhibited varied SARS-CoV-2 neutralization potency with median inhibitory concentration (IC50) values ranging from >10 μg /ml to < 0.001 μg /ml. As of May 2022, 11 monoclonal antibody (mAb) drugs have received the Emergency Use Authorization (EUA). However, the continued emergence of SARS-CoV-2 variants with marked immune escape is becoming the next major challenge in the fight against the global pandemic [18,19]. Thus, it is timely to review SARS-CoV-2 antibody neutralizing mechanisms to inform the next-generation development of antibody and vaccine pharmaceuticals with strong resistance to SARS-CoV-2 immune escape. The benefits of that review will include (1) gaining a deep understanding of antibody-based therapeutics for COVID-19 and other infectious diseases, especially those caused by respiratory viruses; (2) understanding how current COVID-19 vaccines afford immunological protection for humans; (3) informing further therapeutic antibody development and guiding the use of available COVID-19 antibodies worldwide; and (4) optimizing current vaccine design strategies and guiding universal vaccine development to fight against emerging SARS-CoV-2 variants.

SARS-CoV-2 neutralizing antibodies are derived from COVID-19 convalescent patients, phage display, naïve libraries, or other strategies [20]. The neutralizing antibodies can be classified into three sets targeting the receptor-binding domain (RBD), the N-terminal domain (NTD), or S2 subunit of SARS-CoV-2 spike (S) glycoprotein [20,21] (Figure 2A). RBD-directed neutralizing antibodies can be further divided into six groups (group A-F) [22,23]. Group A neutralizing antibodies are mainly encoded by IGHV3-53 and IGHV3-66 germline genes. One representative antibody is CB6 that has obtained emergency use authorization. Group B neutralizing antibodies are frequently encoded by IGHV1-58. Those antibodies such as AZD8895 usually bind to the left shoulder of the RBD (Figure 2B). The neutralizing antibodies encoded by IGHV1-2 and IGHV1-69 are enriched in group C. They mostly bind to the right shoulder of the RBD (Figure 2B). A prominent member in this group is LY-CoV555. Group D neutralizing antibodies are derived from diverse germline genes. Prominent RGEN10987 and AZD1061 belong to this group. The epitopes of group A-D neutralizing antibodies usually overlap with RBD residues that are involved in binding to hACE2 (Figure 2B). SARS-CoV-2 S glycoproteins can undergo a hinge-like movement to transition between “up” and “down” conformations [24-26]. hACE2 can only engage with RBD in the “up” conformation [27]. Most of group A and B neutralizing antibodies can only recognize “up” RBD, whereas most of group C and D neutralizing antibodies recognize both “up” and “down” RBDs. Group E and F neutralizing antibodies, represented by S309 and CR3022, respectively, target outside the hACE2-binding site so that they rarely compete with hACE2 [28] (Figure 2B). Based on the neutralizing antibodies, we outline the following five neutralizing mechanisms.

## Blocking SARS-CoV-2 binding to hACE2 via binding-site competition

**Figure 1.**
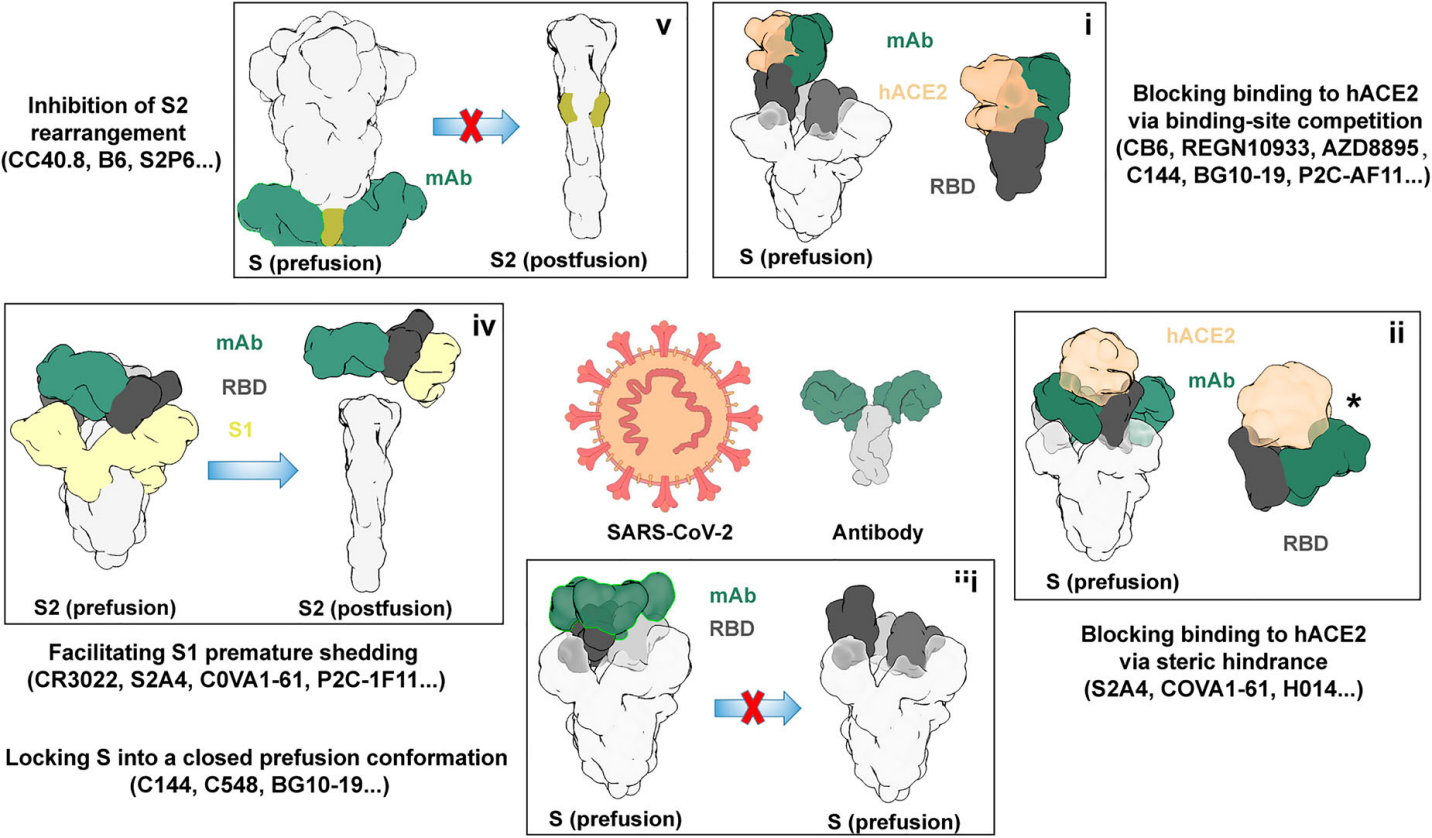
Summary of SARS-CoV-2 antibody neutralizing mechanisms.

In SARS-CoV-2 S glycoprotein, RBD within the S1 subunit is responsible for engaging with the host hACE2 and initiating virus infection [3,15,29]. In RBD, the receptor-binding motif (RBM) is shown to be immunodominant following SARS-CoV-2 infection or vaccine immunizations but also variable over virus evolution [27,30]. Many mAbs that recognize different epitopes fully or partially overlapping with RBM have been developed. They exert potent neutralizing activity against SARS-CoV-2 by blocking RBD binding to hACE2 and preventing viral attachment to host cells [31-34] (Figure 1). Group A-D neutralizing antibodies belong to RBM antibodies [22]. Among the 11 mAbs EUA received, except S309, all the others belong to RBM antibodies with excellent SARS-CoV-2 neutralizing potency. However, as mentioned above, the RBM region is a hotspot for mutations as observed in coronaviruses and SARS-CoV-2 variants [27,35-37]. As a result, the majority of RBM antibodies have a narrow neutralizing breadth among coronaviruses, and many mutations within RBM that have occurred in SARS-COV-2 variants of concern (VOCs) such as E484K, K417N and E484N abrogated the binding to RBD and thus enabled neutralization escape from those antibodies [4,27,38-41]. However, although relatively rare, several RBM mAbs, including S2E12, A23-58.1, 87G7, and LY-CoV1404, which exhibited both potent and broad neutralizing activities against a diverse panel of SARS-CoV-2 variants, were successfully screened out [31,42-44].

**Figure 2.**
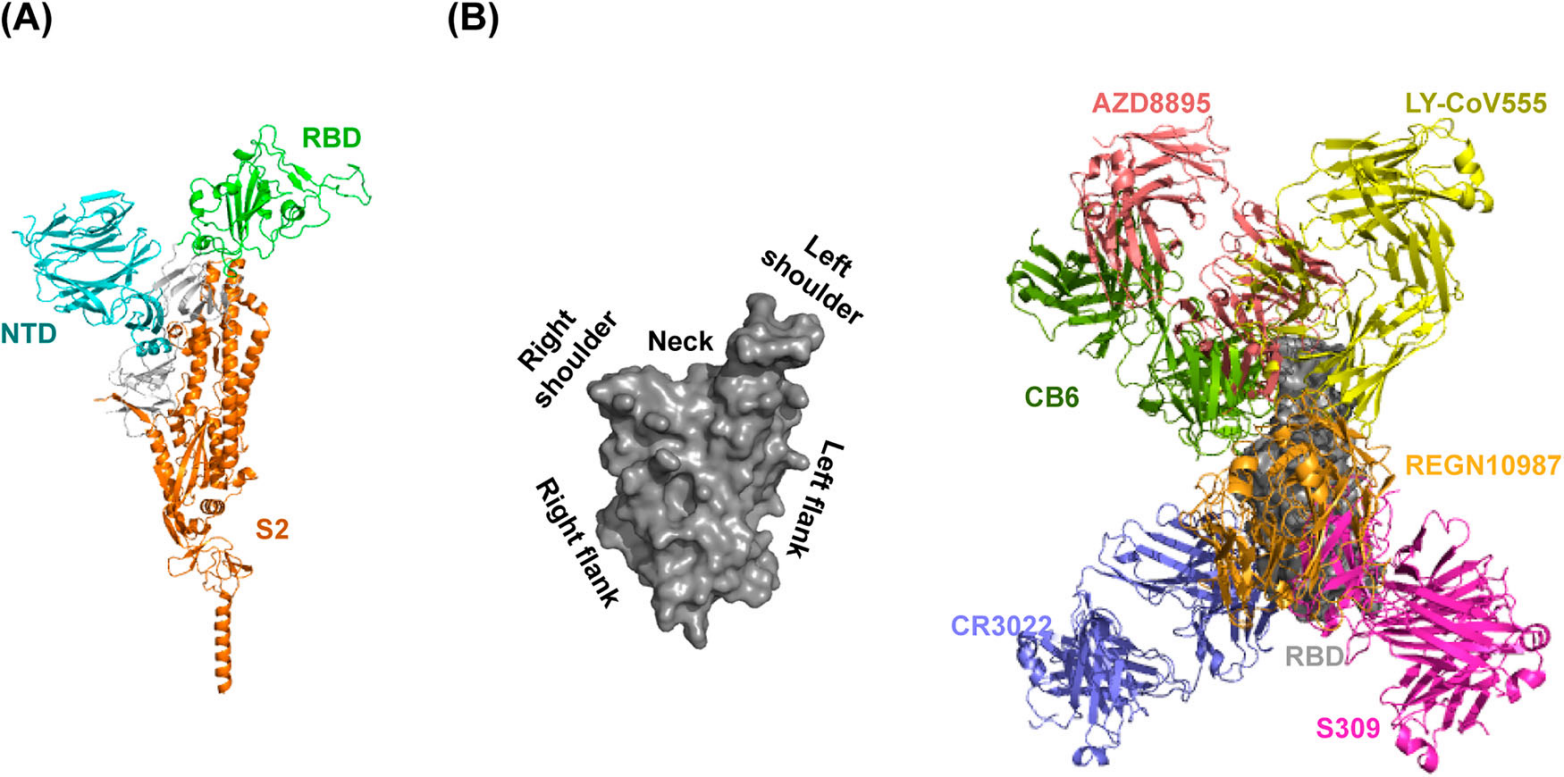
Neutralizing antibody-targeted regions in SARS-CoV-2 spike glycoprotein. (A) Overall crystal structure of monomeric spike with RBD in the closed conformation (PDB: 6XR8) and (B) RBD surface torso analogy in left; representative antibody structures of RBD epitope group A (CB6, PDB: 7C01), B (AZD8897, PDB: 7L7E), C (LY-CoV555, PDB: 7KMG), D (REGN10987, PDB: 6XDG), E (S309, PDB: 7R6X), and F (CR3022, PDB: 6XC7) in right.

**Figure 3.**
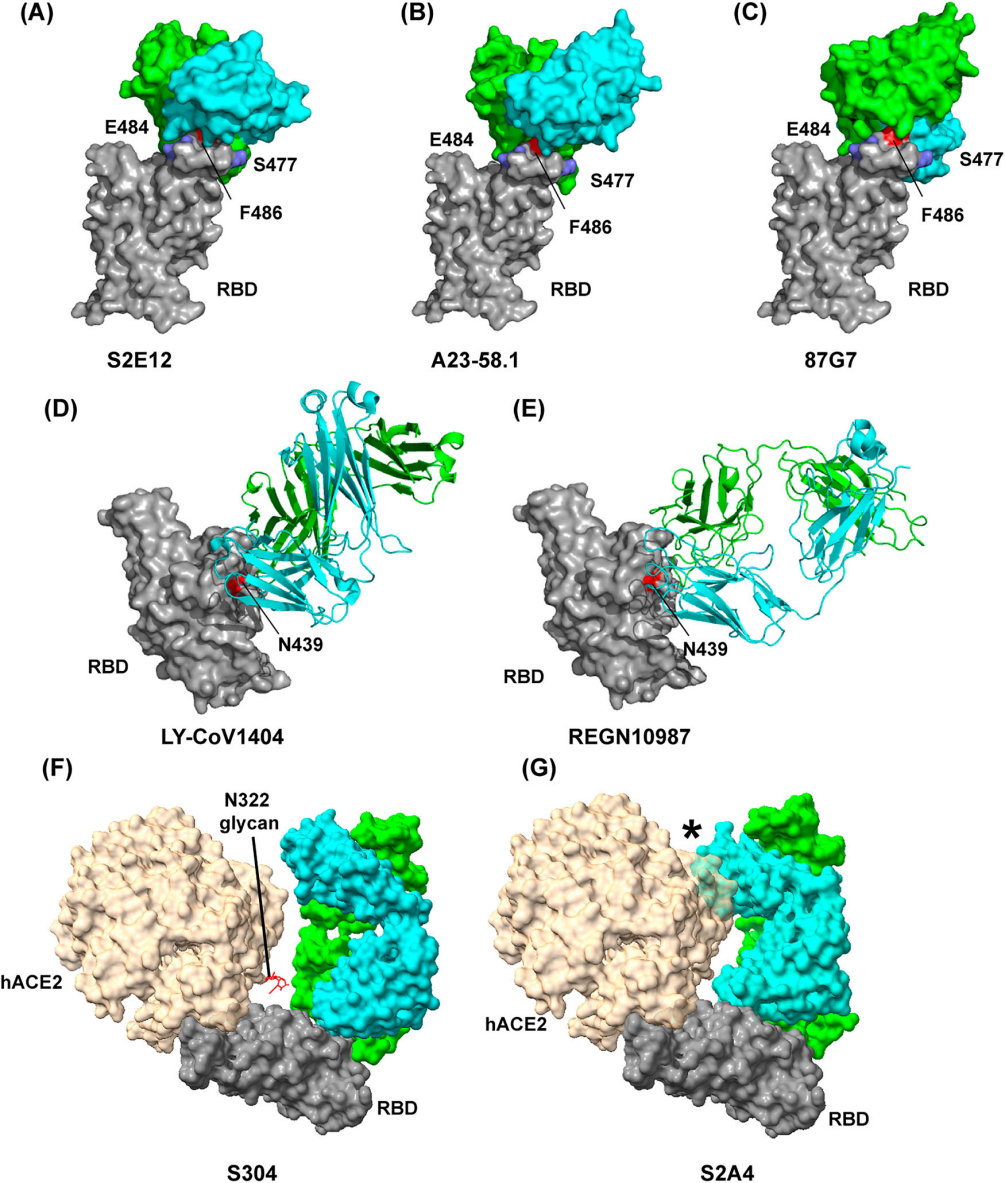
Structures of neutralizing antibody bound to SARS-CoV-2 RBD protein. Footprint of the RBD protein bound to the (A) S2E12 antibody (PDB:7K45); (B) A23-58.1 antibody (PDB: 7LRS); (C) 87G7 antibody (PDB: 7R40); (D) LY-CoV1404 antibody (PDB: 7MMO); (E) REGN10987 antibody (PDB: 6XDG); (F) S304 antibody (PDB: 7JW0); and (G) S2A4 antibody (PDB: 7JVA). In (A), (B) and (C), RBD residues E484 and S477 are indicated as blue, and residue F486 is indicated as red. In (D) and (E), RBD residue N439 is indicated as red. In (F) and (G), the positioning of hACE2 (PDB: 7FDG) that is relative to the antibody Fab bound to the RBD protein is indicated. hACE2 N-linked glycan at position N322 is indicated in (F). The black star indicates steric clashes in (G).

S2E12 and A23-58.1 are derived from the same IGHV1-58 and IGKV 3–20 germlines, display high sequence identity and have highly similar RBD-binding modes [31] (Figure 3A-B). Structural analysis revealed that they bind parallel to the longest axis of the hACE2 binding site and extensively pack the hACE2-contact RBD F486 within a cavity formed by aromatic residues at the antibody light-heavy-chain interface, and mutation-prone residues E484 and S477 are located at the epitope periphery [31] (Figure 3A-B). Both antibodies can potently neutralize Alpha, Beta, Gamma, and Delta, but not Omicron, and showed a certain degree of neutralization breadth [31]. 87G7 has the IGHV3-23 and IGKV3-11 germline origins and also belongs to F486-targeting antibodies [43] (Figure 3C). However, 87G7 has distinct binding features (Figure 3A-C). It is oriented perpendicularly to the receptor-binding ridge with a rotation of 122 degrees relative to S2E12 and A23-58.1[43] (Figure 3A-C). 87G7 retains potent neutralizing activity against all currently known VOCs of SARS-CoV-2, including Omicron BA.1 and BA.2 [43].

Like 87G7, LY-CoV1404 is another RBM-targeting antibody that can potently neutralize Omicron (BA.1 and BA.2.) and other SARS-CoV-2 VOCs [42]. Of note, the approved LY-CoV1404 is the only effective mAb therapeutic option to combat the current Omicron pandemic. Structure determination revealed that LY-CoV1404 is a class 2 antibody, and its binding epitope is very similar to REGN10987 [42] (Figure 3D-E). However, unlike REGN10987 mainly utilizes its heavy chain to bind to RBD, LY-CoV1404 engages the RBD through its heavy and light chains [42,45] (Figure 3D-E). Among RBD residues with which LY-CoV1404 interacts, only N439 and N501 are sensitive to mutations that have arisen to date. Interestingly, the N439K mutant nullified the activity of neutralizing REGN10987, whereas LY-CoV1404 retained full neutralizing potency against a pseudovirus with the N439K mutant, indicating that N439 does not play a critical role in LY-CoV1404 binding to RBD [42]. Those data highlight that even within the evolutionarily plastic RBM, some epitopes still enable robustness of antibodies to viral escape.

## Blocking SARS-CoV-2 binding to hACE2 via steric hindrance

Because of binding-site competition, RBM antibodies block SARS-CoV-2 binding to the hACE2 receptor [36]. In contrast, some mAbs from group F antibodies, which target a cryptic and conserved epitope distinct from the hACE2-binding site [28], can compete with the hACE2 receptor to engage SARS-CoV-2 RBD because of a steric hindrance rather than binding-site competition [45-47] (Figure 1). Structure investigation reveals that COVA1-16, REGN10985, S2X35, S2A4, and S304 show steric clashes with hACE2 to various extents upon binding to RBD [46-48] (Figure 3F-G) and antibody neutralization potencies positively correlates with the steric hindrance extent [47], implying that blocking virus binding to host receptor is a very effective or vital mechanism for SARS-CoV-2 antibody neutralization. For example, S304 shows weak steric hindrance with hACE2 N322 glycan, whereas S2A4 has a substantial steric clash with hACE2 and strongly hinder hACE2 binding [47] (Figure 3F-G). As a result, S2A4 is a more potent neutralizer than S304 [47]. However, it is notable that not all group F antibodies can block binding to hACE2 by steric hindrance. For example, CR3022 and hACE2 can simultaneously engage SARS-CoV-2 RBD without any clashing [49]. Other neutralizing mechanisms will be discussed in detail below.

S309, a group E antibody, targets an epitope that is exposed in both “up” and “down” RBD and distal from the RBM region, and it is expected to have access to all three epitopes on the S trimer [50]. Neutralization assays showed that S309 Fab displayed a marked potency decrease in terms of maximal neutralization plateau (80%) reached, as compared to IgGs (100%) [50]. An Fc-silenced version of S309 (GH-S309-N297A) has been shown to confer similar protection against SARS-CoV-2 infection in Syrian hamsters as native S309 [51], suggesting that the neutralizing activity of S309 is the primary protective mechanism. In addition, S309 IgG effectively blocked the hACE2-dependent entry of SARS-CoV-2 in the presence of membrane lectins [51]. Thus, high-density S309 IgG binding on virions may block SARS-CoV-2 binding to host hACE2 via steric hindrance [10]. Similar to S309, some NTD-specific mAbs including S2L28, S2M28, S2X28, and S2X333 showed an increased neutralization potency in the IgG formats than in their Fab formats, indicating that the steric hindrance provided by Fc positioning may at least partly contribute to their neutralizing activity [52].

## Locking S into a closed prefusion conformation

Some antibodies can lock all three RBDs of S into a closed prefusion conformation and thus render S unable to open to bind hACE2 [10,53,54] (Figure 1). Antibodies that utilize this neutralizing mechanism can be further divided into several categories based on different antibody domains utilized to lock RBDs into “down” conformation as exemplified by studies with C144, C548 and BG10-19 [28,55,56].

**Figure 4.**
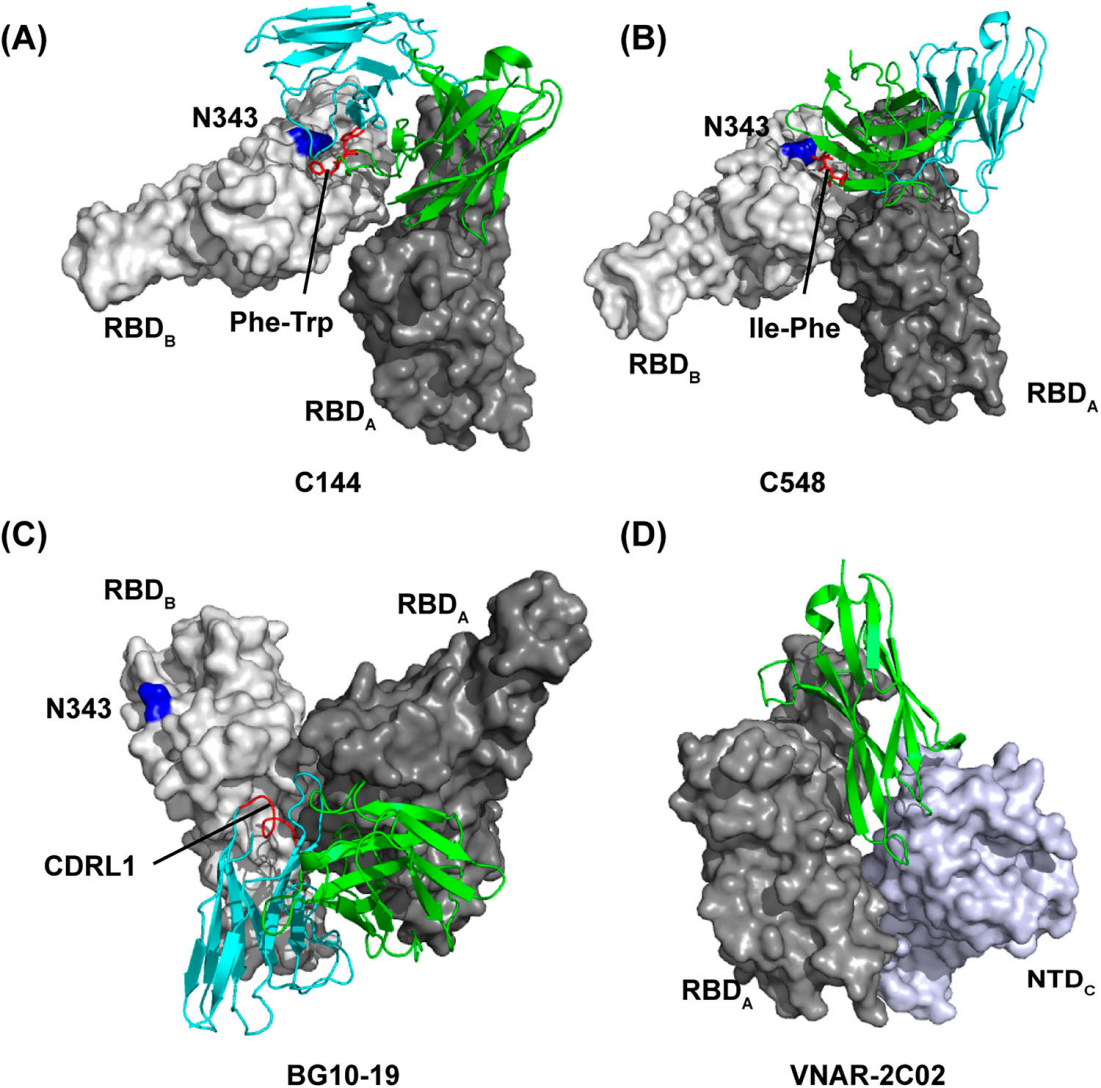
Distinct binding modes of the antibody-S complex lock S into “down” prefusion conformation. Close-up view of quaternary epitope of the (A) C144 antibody (PDB:7K90); (B) C548 antibody (PDB: 7R8O); (C) BG10-19 antibody (PDB: 7M6E); and (D) VNAR-2C02 antibody (PDB: 7SPP) involving bridging between adjacent promoters. In (A), (B), and (C), residue N343 in adjacent RBD is indicated as blue, and the antibody critical residues or loop in binding to adjacent RBD are indicated as red.

C144 is a public VH3-53-encoded long CDRH3 human mAb and belongs to class 2 antibodies [28]. Although the binding epitope would be accessible on “up” RBDs, all C144-bound S trimers exhibit a unique completely closed conformation with three “down” RBDs [28]. Cryo-EM structure revealed that C144 inserts Phe-Trp at the tip of the long CDRH3 to a hydrophobic patch near the N343-glycan base on the adjacent RBD to bridge between adjacent “down” RBDs (Figure 4A), and further stabilizes the S trimer into a closed prefusion conformation [28]. C548 is a human VH1-69-encoded class 2 antibody, which encodes hydrophobic I53-F54 residues at the tip of CDRH2. Similar to C144, those hydrophobic residues target the hydrophobic patch on the adjacent RBD to lock RBDs into “down” conformation [56] (Figure 4B). In contrast, BG10-19 is a group E mAb exhibiting strong neutralizing potency against both SARS-CoV-2 and SARS-CoV. It binds a primary RBD epitope outside the hACE2 receptor-binding motif by CDRH1-3 and CDRL2 loops like S309 [55] (Figure 4C). Notably, by positioning its CDRL1 loop atop a neighbouring “down” RBD, BG10-19 can also lock the S trimer into a closed prefusion conformation [55] (Figure 4C). It should be noted that unlike the bridging mode of C144 and C548, BG10-19 does not target the above-mentioned hydrophobic patch on the adjacent RBD (Figure 4A-C). In addition, VNAR-2C02, a variable new antigen receptor from the immune system of sharks, was reported to pin the “down” RBD and adjacent NTD together to prevent the RBD from adopting the “up” conformation [57] (Figure 4D). These data demonstrated that antibodies utilize distinct binding modes although sharing a convergent neutralizing mechanism of locking RBDs into the “down” position.

## Facilitating S1 premature shedding

The binding of SARS-CoV-2 RBD to host hACE2 weakens the S1-S2 interface, likely by decreasing the buried surface area at the interface, and further triggers S1 shedding from S2 and irreversible refolding of S2 into a post-fusion conformation, which provides free energy for viral and host membrane fusion [25,58]. For this process, an “up” RBD in S is necessary [25]. Some mAbs can neutralize SARS-CoV-2 by similarly triggering S1 premature shedding and thus rendering S non-functional for viral entry (Figure 1).

**Figure 5.**
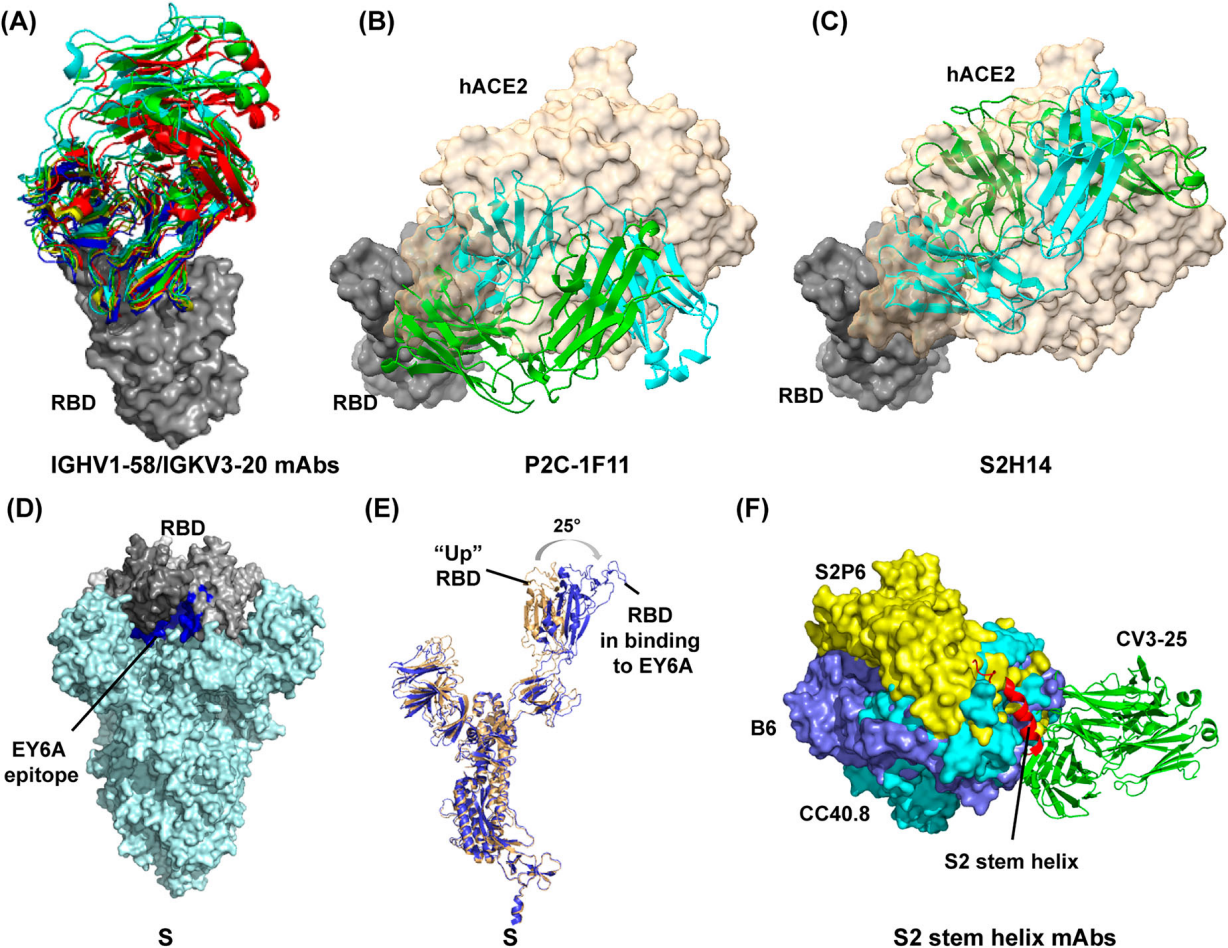
Structures of antibodies bound to RBD or S2 stem helix peptide and RBD conformational change following binding to a group F antibody (EY6A). The footprint of the RBD protein bound to the (A) IGHV1-58/IGKV3-20 public antibodies; (B) P2C-1F11 antibody (PDB: 7CDI); and (C) S2H14 antibody (PDB: 7JX3). In (A), the antibody 253 (PDB: 7BEN), 58G6 (PDB: 7E3L), BD836 (PDB: 7EZV), S2E12 (PDB: 7K45), and A23-58.1 (PDB: 7LRT) is indicated as red, green, blue, yellow, and cyan, respectively. In (B) and (C), hACE2 (PDB: 7FDG) binding to RBD relative to Fab-RBD binding is indicated. (D) EY6A epitope is inaccessible in S protein whose RBDs adopt “down” conformation. EY6A epitope is indicated in blue. (E) Comparing the EY6A-bound S structure with an open-form S structure (PDB: 6VYB). (F) The footprint of SARS-CoV-2 S2 stem helix bound to the antibody S2P6 (PDB: 7RNJ), B6 (PDB: 7M53), CC40.8 (PDB: 7SJS), and CV3-25 (PDB: 7NAB).

A cluster of IGHV1-58/IGKV3-20 public antibodies, including S2E12, 253, CV3-1, A23-58.1, BD-836, 58G6, and CoV2-2196, have low levels of somatic hypermutation with potent neutralizing activity [44,53,59-65]. They are only accessible to “up” RBDs in S, belong to group B antibodies, and bind the RBD left shoulder with an identical angle [59] (Figure 5A). For these antibodies, CDRH1-3 and CDRL1-3 participate in the long CDRH3-dominated paratope (Figure 5A), and germline-encoded residues mediate nearly all contacts with RBD. Among them, CV3-1 has demonstrated that antibody incubation rendered the virus to lose most prefusion S proteins and thus to exhibit S in post-fusion conformation [59]. In SARS-CoV-2 S-expressed cell-based assays, CV3-1 displayed a superior potency of releasing S1 into cell culture supernatant compared to hACE2 [59]. Those data strongly indicated that CV3-1 could exert SARS-CoV-2 neutralization efficacy by potently triggering S1 premature shedding [59]. Although other IGHV1-58/IGKV3-20 mAbs remain to be further investigated, the high similarities of residue sequence and binding mode imply sharing of the same neutralizing mechanism of triggering S1 premature dissociation from S2. P2C-1F11, one IGHV3-66 public antibody, has also been reported as a potent agonist for S1 premature shedding following its binding to RBD [66]. Notably, structural analysis revealed that P2C-1F11 also only binds to RBD in “up” conformation but unlike IGHV1-58/IGKV3-20 antibodies, it targets both the RBD left shoulder and the neck region with high mimicry of hACE2 binding [66] (Figure 5B). In addition, another antibody, S2H14, is encoded by IGHV3-15 and IGLV5-57 with no somatic hypermutations in variable regions [47]. Like P2C-1F11, S2H14 targets an epitope largely overlapping with hACE2-binding sites in the RBM (Figure 5B-C) and can promote S1 premature shedding from cell-surface-expressed spike proteins [47]. Clearly, not all antibodies that preferentially recognize RBD in “up” conformation as hACE2 can exert neutralizing activity by triggering S1 shedding, and further mechanism studies regarding how S1 evolved to be activated by the hACE2 receptor will help identify potential antibodies that can act to trigger S1 shedding.

Another antibody cluster capable of triggering S1 shedding is from group F antibodies and includes CR3022, S2A4, S304, S2X35, EY6A and COVA1-16 [46,47,49,67-69]. As mentioned above, these antibodies target conserved and cryptic epitopes in the left flank of RBD, which are away from the hACE2 binding sites and completely buried in S glycoprotein when RBD is in the “down” conformation [49] (Figure 5D). Cryo-EM analysis reveals that even when the targeted RBD in S adopts the “up” conformation, antibody Fabs still clash with the adjacent “down” RBD, S2 subunit and NTD domain [49,68]. Thus, to bind to these antibodies, at least two RBDs in the S glycoprotein are needed to simultaneously adopt the “up” configuration [67,68,70]. Moreover, to relieve clashes with adjacent NTD domain and S2 subunit, antibody binding usually further forces the RBD to rotate outwards to various extent, for example by 25° in EY6A, such that RBD seems to be more open than its “up” conformation in hACE2-binding mode [49,68] (Figure 5E), and presumably leading to a more destabilized state of the S1-S2 interface. In line with that, all the antibodies (CR3022, S2A4, S304, S2X35, and EY6A) were confirmed to trigger S1 premature shedding potently, as evidenced by Cryo-EM data which showed that many S glycoproteins lost structural integrity on incubation with these antibodies [47,49,68].

Notably, one group E antibody, Beta-53, targeting the right flank of RBD, also exhibited a potency of S destruction following its incubation with S and thus may exert neutralizing activity in part by triggering S1 shedding [71]. However, more detailed information is lacking. Several nanobodies, such as Nb17 and VHH E, were demonstrated to induce aberrant activation of the fusion machinery [72,73]. One antibody against SARS-CoV, S230, was also found to trigger the SARS-CoV spike transition from prefusion to post-fusion conformation [74]. Therefore, premature activation of spike conformational changes and further inactivation of the fusion machinery by antibody engagement appear to be a universal neutralizing mechanism for antibodies against coronaviruses.

As mentioned above, some group F antibodies (distant from hACE2-binding sites) and those hACE2-mimicry antibodies (largely overlapping with hACE2-binding sites) can bind RBD non-competitively, but both induce S1 premature shedding, theoretically leading to neutralization synergy and thus a cocktail of antibodies from those two clusters seem to be rational.

## S2 destruction and prevention of S2 conformational change

Upon engaging the S1 subunit of S glycoprotein with hACE2, the S2 subunit fusion machinery undergoes a markedly conformational rearrangement to mediate the fusion of host cell and virus membranes [75]. As a part of the S2 fusion machinery, the S2 stem helix region (residues 1140–1160) is somewhat immunogenic, and several NAbs, including S2P6, CC40.8, IgG22, B6, 28D9, and CV3-25, have been isolated from convalescent patients, vaccine recipients or mice [59,76-80]. Consistent with the high conservation of the S2 stem helix epitope among betacoronavirus, those NAbs usually exhibit a great neutralization breadth against betacoronavirus, including SARS-CoV-1 and SARS-CoV-2 [76,77]. Because of the high flexibility of the epitope in structure, a high-resolution helix-fab structure in the S context has not been achieved. However, the details on antibody binding can be obtained from the crystal structure of antibody Fab in complex with the stem helix peptide.

The structural investigation reveals that B6, S2P6, and CC40.8 mainly interact with the N-terminal a-helix of the epitope [76,77,79], whereas CV3-25 targets both the a-helix and the C-terminal loop [59] (Figure 5F). These antibodies would clash with the adjacent protomer when bound to the S glycoprotein in the prefusion conformation [77]. They may induce disruption of the S2 stem quaternary organization to relieve the clash. This hypothesis was supported by the observed much stronger binding of CC40.8 to S2P than to S6P, where four additional proline substitutions in S2 subunit are added to stabilize the S trimer in prefusion conformation [59]. Moreover, during the S transition from the prefusion to post-fusion conformation, the stem helix epitope undergoes a large conformational change and is eventually buried at the interface with the other two promoters from the S trimer [76] (Figure 1). Thus, in theory, antibody binding to prefusion S should sterically hinder the conformational change. Of note, the conserved S2 stem helix epitope seems linear and can be independently expressed in vitro [59], which offers easy access to the investigation of its potential as an immunogen in vaccine development.

## Other potential SARS-CoV-2 neutralizing mechanisms

Except for the above five neutralization mechanisms with robust evidence from Cryo-EM data or cell-based assays, there are some other potential or presumable mechanisms. Modified neutralization assays with antibody incubation after the virus absorbed cells demonstrated that two NTD-targeting mAbs, COV2-2489 and COV2-2676, can neutralize SARS-CoV-2 at a post-attachment step [81]. However, a more detailed neutralizing mechanism remains to be further investigated. Another NTD-specific mAb, C1717, targets the viral membrane-proximal side of NTD, and its light chain is close to the fusion peptide of the S2 subunit [82]. Authors speculate that the proximity to the S2 fusion machinery may contribute to antibody neutralizing activity by preventing enzyme access to the S2 cleavage site [82]. In addition, one RBD-directed antibody, 5A6, was demonstrated in cell-based assays to exert neutralizing activity by inhibiting S-mediated syncytia formation [69]. However, the detailed molecular and structural mechanism remains unclear. Although playing no role in antibody neutralization in vitro, Fc-dependent effector mechanisms, antibody-dependent cytotoxicity (ADCC) by natural killer cells and antibody-dependent cellular phagocytosis (ADCP) by macrophages or dendritic cells, can contribute to a protective effect against SARS-CoV-2 infection in vivo by clearing the virus and infected cells and by stimulating a T cell response [83,84]. S309 and some NTD-specific mAbs such as S2L28, S2M28, S2X28, and S2X33 have been demonstrated to mediate strong ADCC and ADCP responses [52].

## Discussion

Continued study on an anti-SARS-CoV-2 antibody provides an extraordinary opportunity to understand how host antibody neutralizes virus, especially coronavirus or respiratory virus. Although we outlined five relatively clear neutralization mechanisms, the full picture remains elusive, and much remains to be further investigated. Notably, regarding one antibody, it may utilize multiple neutralizing mechanisms. For example, on the one hand, S2A4 can neutralize SARS-CoV-2 by blocking virus binding to the hACE2 receptor via steric hindrance; on the other hand, it can also promote S1 premature shedding and thus render S to be non-functional for viral entry [47]. Group A-D antibodies share a common neutralizing mechanism of blocking SARS-CoV-2 binding to the host hACE2 receptor via binding-site competition [28]. In the meantime, some mAbs can lock S into a closed prefusion conformation (C144) or promote S1 premature shedding (P2C-1F11) to exert SARS-CoV-2-neutralizing activity [28,66]. Moreover, some mAbs seem to interfere with each other in neutralizing the virus, leading to additional advantages or disadvantages from their cocktail. For instance, the antibody such as C144, C548, or BG10-19 which can lock all RBDs of S into “down” conformation can possibly nullify the activity of group F antibodies such as S304, whose cryptic epitopes are completely inaccessible on “down” RBDs [28,49]. Some RBM antibodies that can promote S1 premature shedding and S transition to post-fusion state as hACE2 mimicry has been recently shown to enhance S-mediated syncytia formation [51] and, therefore, possibly stand a risk of facilitating virus cell-to-cell spread. In theory, the S2 stem helix-targeted mAb may lower this risk by preventing S transition to post-fusion conformation and cell syncytia formation. In line with the analysis, syncytia formation induced by S2E12 was indeed inhibited by S2P6 [51]. Therefore, to eliminate potential safety risks, combining an S2E12-like antibody with one S2 stem helix-targeted mAb appears to be rational.

The ongoing SARS-CoV-2 evolution that causes a marked decrease in neutralization potency of sera from vaccine recipients highlights the importance of developing next-generation vaccines with robustness to viral mutations [85-87]. Fortunately, there are various conserved epitopes on S trimers such as those targeted by LY-CoV1404, S2A4, S309, and S2P6. However, the low frequencies of those broad neutralizing antibodies in individuals suggest that such conserved epitopes seem to be subdominant following infection or vaccine immunizations [42,76,88]. Thus, next-generation SARS-CoV-2 universal vaccines should seek to improve immune response to those conserved epitopes possibly by unmasking them. Of note, as group F antibody epitopes are usually deeply buried within the S trimer theoretically, it is an RBD-based rather than an S-based vaccine immunogen that has the potential of eliciting a larger number of such cross-neutralization antibodies. Consistent with that, compared with the whole virus- or S-based vaccine, the RBD-based vaccine exhibits increased resistance to SARS-CoV-2 variants [89,90].

## References

[1] Altmann DM, Boyton RJ. COVID-19 vaccination: The road ahead. Science (New York, NY). 2022 Mar 11;375(6585):1127–1132. doi:10.1126/science.abn1755. 35271316

[2] Dai L, Gao GF. Viral targets for vaccines against COVID-19. Nat Rev Immunol. 2021 Feb;21(2):73–82. doi:10.1038/s41577-020-00480-0. 33340022 PMC7747004

[3] Huang Q, Ji K, Tian S, et al. A single-dose mRNA vaccine provides a long-term protection for hACE2 transgenic mice from SARS-CoV-2. Nat Commun. 2021 Feb 3;12(1):776. doi:10.1038/s41467-021-21037-2. 33536425 PMC7858593

[4] Abu-Raddad LJ, Chemaitelly H, Butt AA. Effectiveness of the BNT162b2 Covid-19 vaccine against the B.1.1.7 and B.1.351 variants. N Engl J Med. 2021 Jul 8;385(2):187–189. doi:10.1056/NEJMc2104974. 33951357 PMC8117967

[5] Dai L, Zheng T, Xu K, et al. A universal design of betacoronavirus vaccines against COVID-19, MERS, and SARS. Cell. 2020 Aug 6;182(3):722–733.e11. doi:10.1016/j.cell.2020.06.035. 32645327 PMC7321023

[6] Baden LR, El Sahly HM, Essink B, et al. Efficacy and safety of the mRNA-1273 SARS-CoV-2 vaccine. N Engl J Med. 2021 Feb 4;384(5):403–416. doi:10.1056/NEJMoa2035389. 33378609 PMC7787219

[7] Heath PT, Galiza EP, Baxter DN, et al. Safety and efficacy of NVX-CoV2373 Covid-19 vaccine. N Engl J Med. 2021 Sep 23;385(13):1172–1183. doi:10.1056/NEJMoa2107659. 34192426 PMC8262625

[8] Tanriover MD, Doğanay HL, Akova M, et al. Efficacy and safety of an inactivated whole-virion SARS-CoV-2 vaccine (CoronaVac): interim results of a double-blind, randomised, placebo-controlled, phase 3 trial in Turkey. Lancet (London, England). 2021 Jul 17;398(10296):213–222. doi:10.1016/s0140-6736(21)01429-x. PMC8266301 34246358

[9] Khoury DS, Cromer D, Reynaldi A, et al. Neutralizing antibody levels are highly predictive of immune protection from symptomatic SARS-CoV-2 infection. Nat Med. 2021 Jul;27(7):1205–1211. doi:10.1038/s41591-021-01377-8. 34002089

[10] Corti D, Purcell LA, Snell G, et al. Tackling COVID-19 with neutralizing monoclonal antibodies. Cell. 2021 Jun 10;184(12):3086–3108. doi:10.1016/j.cell.2021.05.005. 34087172 PMC8152891

[11] Gruell H, Vanshylla K, Weber T, et al. Antibody-mediated neutralization of SARS-CoV-2. Immunity. 2022, doi:10.1016/j.immuni.2022.05.005. PMC9118976 35623355

[12] Jones BE, Brown-Augsburger PL, Corbett KS, et al. The neutralizing antibody, LY-CoV555, protects against SARS-CoV-2 infection in nonhuman primates. Sci Transl Med. 2021 May 12;13(593), doi:10.1126/scitranslmed.abf1906. PMC8284311 33820835

[13] Baum A, Ajithdoss D, Copin R, et al. REGN-COV2 antibodies prevent and treat SARS-CoV-2 infection in rhesus macaques and hamsters. Science (New York, NY). 2020 Nov 27;370(6520):1110–1115. doi:10.1126/science.abe2402. PMC7857396 33037066

[14] Kim C, Ryu DK, Lee J, et al. A therapeutic neutralizing antibody targeting receptor binding domain of SARS-CoV-2 spike protein. Nat Commun. 2021 Jan 12;12(1):288. doi:10.1038/s41467-020-20602-5. 33436577 PMC7803729

[15] Shi R, Shan C, Duan X, et al. A human neutralizing antibody targets the receptor-binding site of SARS-CoV-2. Nature. 2020 Aug;584(7819):120–124. doi:10.1038/s41586-020-2381-y. 32454512

[16] Ju B, Zhang Q, Ge J, et al. Human neutralizing antibodies elicited by SARS-CoV-2 infection. Nature. 2020 Aug;584(7819):115–119. doi:10.1038/s41586-020-2380-z. 32454513

[17] Hansen J, Baum A, Pascal KE, et al. Studies in humanized mice and convalescent humans yield a SARS-CoV-2 antibody cocktail. Science (New York, NY). 2020 Aug 21;369(6506):1010–1014. doi:10.1126/science.abd0827. PMC7299284 32540901

[18] Tarke A, Coelho CH, Zhang Z, et al. SARS-CoV-2 vaccination induces immunological T cell memory able to cross-recognize variants from alpha to omicron. Cell. 2022 Mar 3;185(5):847–859.e11. doi:10.1016/j.cell.2022.01.015. 35139340 PMC8784649

[19] Uraki R, Kiso M, Iida S, et al. Characterization and antiviral susceptibility of SARS-CoV-2 omicron/BA.2. Nature. 2022 May 16, doi:10.1038/s41586-022-04856-1. PMC10579982 35576972

[20] Hastie KM, Li H, Bedinger D, et al. Defining variant-resistant epitopes targeted by SARS-CoV-2 antibodies: A global consortium study. Science (New York, NY). 2021 Oct 22;374(6566):472–478. doi:10.1126/science.abh2315. PMC9302186 34554826

[21] Kombe Kombe AJ, Zahid A, Mohammed A, et al. Potent molecular feature-based neutralizing monoclonal antibodies as promising therapeutics against SARS-CoV-2 infection. Front Mol Biosci. 2021;8:670815. doi:10.3389/fmolb.2021.670815. 34136533 PMC8201996

[22] Cao Y, Wang J, Jian F, et al. Omicron escapes the majority of existing SARS-CoV-2 neutralizing antibodies. Nature. 2022 Feb;602(7898):657–663. doi:10.1038/s41586-021-04385-3. 35016194 PMC8866119

[23] Yuan M, Huang D, Lee CD, et al. Structural and functional ramifications of antigenic drift in recent SARS-CoV-2 variants. Science (New York, NY). 2021 Aug 13;373(6556):818–823. doi:10.1126/science.abh1139. PMC8284396 34016740

[24] Cai Y, Zhang J, Xiao T, et al. Distinct conformational states of SARS-CoV-2 spike protein. Science (New York, NY). 2020 Sep 25;369(6511):1586–1592. doi:10.1126/science.abd4251. PMC7464562 32694201

[25] Wrapp D, Wang N, Corbett KS, et al. Cryo-EM structure of the 2019-nCoV spike in the prefusion conformation. Science (New York, NY). 2020 Mar 13;367(6483):1260–1263. doi:10.1126/science.abb2507. PMC7164637 32075877

[26] Lv Z, Deng YQ, Ye Q, et al. Structural basis for neutralization of SARS-CoV-2 and SARS-CoV by a potent therapeutic antibody. Science (New York, NY). 2020 Sep 18;369(6510):1505–1509. doi:10.1126/science.abc5881. PMC7402622 32703908

[27] Dejnirattisai W, Huo J, Zhou D, et al. SARS-CoV-2 omicron-B.1.1.529 leads to widespread escape from neutralizing antibody responses. Cell. 2022 Feb 3;185(3):467–484.e15. doi:10.1016/j.cell.2021.12.046. 35081335 PMC8723827

[28] Barnes CO, Jette CA, Abernathy ME, et al. SARS-CoV-2 neutralizing antibody structures inform therapeutic strategies. Nature. 2020 Dec;588(7839):682–687. doi:10.1038/s41586-020-2852-1. 33045718 PMC8092461

[29] Wang Q, Zhang Y, Wu L, et al. Structural and functional basis of SARS-CoV-2 entry by using human ACE2. Cell. 2020 May 14;181(4):894–904.e9. doi:10.1016/j.cell.2020.03.045. 32275855 PMC7144619

[30] Huang Q, Zeng J, Yan J. COVID-19 mRNA vaccines. J Genet Genomics. 2021 Feb 20;48(2):107–114. doi:10.1016/j.jgg.2021.02.006. 34006471 PMC7959685

[31] Starr TN, Czudnochowski N, Liu Z, et al. SARS-CoV-2 RBD antibodies that maximize breadth and resistance to escape. Nature. 2021 Sep;597(7874):97–102. doi:10.1038/s41586-021-03807-6. 34261126 PMC9282883

[32] Du S, Cao Y, Zhu Q, et al. Structurally resolved SARS-CoV-2 antibody shows high efficacy in severely infected hamsters and provides a potent cocktail pairing strategy. Cell. 2020 Nov 12;183(4):1013–1023.e13. doi:10.1016/j.cell.2020.09.035. 32970990 PMC7489885

[33] Yuan M, Liu H, Wu NC, et al. Structural basis of a shared antibody response to SARS-CoV-2. Science (New York, NY). 2020 Aug 28;369(6507):1119–1123. doi:10.1126/science.abd2321. PMC7402627 32661058

[34] Vanshylla K, Fan C, Wunsch M, et al. Discovery of ultrapotent broadly neutralizing antibodies from SARS-CoV-2 elite neutralizers. Cell Host Microbe. 2022 Jan 12;30(1):69–82.e10. doi:10.1016/j.chom.2021.12.010. 34973165 PMC8683262

[35] Cele S, Jackson L, Khoury DS, et al. Omicron extensively but incompletely escapes pfizer BNT162b2 neutralization. Nature. 2022 Feb;602(7898):654–656. doi:10.1038/s41586-021-04387-1. 35016196 PMC8866126

[36] Nabel KG, Clark SA, Shankar S, et al. Structural basis for continued antibody evasion by the SARS-CoV-2 receptor binding domain. Science (New York, NY). 2022 Jan 21;375(6578):eabl6251. doi:10.1126/science.abl6251. PMC9127715 34855508

[37] Wang P, Casner RG, Nair MS, et al. Increased resistance of SARS-CoV-2 variant P.1 to antibody neutralization. Cell Host Microbe. 2021 May 12;29(5):747–751.e4. doi:10.1016/j.chom.2021.04.007. 33887205 PMC8053237

[38] McCallum M, Walls AC, Sprouse KR, et al. Molecular basis of immune evasion by the delta and kappa SARS-CoV-2 variants. Science (New York, NY). 2021 Dec 24;374(6575):1621–1626. doi:10.1126/science.abl8506. PMC12240541 34751595

[39] Mlcochova P, Kemp SA, Dhar MS, et al. SARS-CoV-2 B.1.617.2 delta variant replication and immune evasion. Nature. 2021 Nov;599(7883):114–119. doi:10.1038/s41586-021-03944-y. 34488225 PMC8566220

[40] Wang R, Zhang Q, Ge J, et al. Analysis of SARS-CoV-2 variant mutations reveals neutralization escape mechanisms and the ability to use ACE2 receptors from additional species. Immunity. 2021 Jul 13;54(7):1611–1621.e5. doi:10.1016/j.immuni.2021.06.003. 34166623 PMC8185182

[41] Gobeil SM, Janowska K, McDowell S, et al. Effect of natural mutations of SARS-CoV-2 on spike structure, conformation, and antigenicity. Science (New York, NY). 2021 Aug 6;373(6555), doi:10.1126/science.abi6226. PMC8611377 34168071

[42] Westendorf K, Žentelis S, Wang L, et al. LY-CoV1404 (bebtelovimab) potently neutralizes SARS-CoV-2 variants. Cell Rep. 2022 May 17;39(7):110812. doi:10.1016/j.celrep.2022.110812. 35568025 PMC9035363

[43] Du W, Hurdiss DL, Drabek D, et al. An ACE2-blocking antibody confers broad neutralization and protection against omicron and other SARS-CoV-2 variants of concern. Sci Immunol. 2022 Apr 26: eabp9312. doi:10.1126/sciimmunol.abp9312. 35471062 PMC9097884

[44] Zost SJ, Gilchuk P, Case JB, et al. Potently neutralizing and protective human antibodies against SARS-CoV-2. Nature. 2020 Aug;584(7821):443–449. doi:10.1038/s41586-020-2548-6. 32668443 PMC7584396

[45] Copin R, Baum A, Wloga E, et al. The monoclonal antibody combination REGEN-COV protects against SARS-CoV-2 mutational escape in preclinical and human studies. Cell. 2021 Jul 22;184(15):3949–3961.e11. doi:10.1016/j.cell.2021.06.002. 34161776 PMC8179113

[46] Liu H, Wu NC, Yuan M, et al. Cross-Neutralization of a SARS-CoV-2 antibody to a functionally conserved site Is mediated by avidity. Immunity. 2020 Dec 15;53(6):1272–1280.e5. doi:10.1016/j.immuni.2020.10.023. 33242394 PMC7687367

[47] Piccoli L, Park YJ, Tortorici MA, et al. Mapping neutralizing and immunodominant sites on the SARS-CoV-2 spike receptor-binding domain by structure-guided high-resolution serology. Cell. 2020 Nov 12;183(4):1024–1042.e21. doi:10.1016/j.cell.2020.09.037. 32991844 PMC7494283

[48] Martinez DR, Schäfer A, Gobeil S, et al. A broadly cross-reactive antibody neutralizes and protects against sarbecovirus challenge in mice. Sci Transl Med. 2022 Jan 26;14(629):eabj7125. doi:10.1126/scitranslmed.abj7125. 34726473 PMC8899823

[49] Huo J, Zhao Y, Ren J, et al. Neutralization of SARS-CoV-2 by destruction of the prefusion spike. Cell Host Microbe. 2020 Sep 9;28(3):445–454.e6. doi:10.1016/j.chom.2020.06.010. 32585135 PMC7303615

[50] Pinto D, Park YJ, Beltramello M, et al. Cross-neutralization of SARS-CoV-2 by a human monoclonal SARS-CoV antibody. Nature. 2020 Jul;583(7815):290–295. doi:10.1038/s41586-020-2349-y. 32422645

[51] Lempp FA, Soriaga LB, Montiel-Ruiz M, et al. Lectins enhance SARS-CoV-2 infection and influence neutralizing antibodies. Nature. 2021 Oct;598(7880):342–347. doi:10.1038/s41586-021-03925-1. 34464958

[52] McCallum M, De Marco A, Lempp FA, et al. N-terminal domain antigenic mapping reveals a site of vulnerability for SARS-CoV-2. Cell. 2021 Apr 29;184(9):2332–2347.e16. doi:10.1016/j.cell.2021.03.028. 33761326 PMC7962585

[53] Tortorici MA, Beltramello M, Lempp FA, et al. Ultrapotent human antibodies protect against SARS-CoV-2 challenge via multiple mechanisms. Science (New York, NY). 2020 Nov 20;370(6519):950–957. doi:10.1126/science.abe3354. PMC7857395 32972994

[54] Schoof M, Faust B, Saunders RA, et al. An ultrapotent synthetic nanobody neutralizes SARS-CoV-2 by stabilizing inactive spike. Science (New York, NY). 2020 Dec 18;370(6523):1473–1479. doi:10.1126/science.abe3255. PMC7857409 33154106

[55] Scheid JF, Barnes CO, Eraslan B, et al. B cell genomics behind cross-neutralization of SARS-CoV-2 variants and SARS-CoV. Cell. 2021 Jun 10;184(12):3205–3221.e24. doi:10.1016/j.cell.2021.04.032. 34015271 PMC8064835

[56] Muecksch F, Weisblum Y, Barnes CO, et al. Affinity maturation of SARS-CoV-2 neutralizing antibodies confers potency, breadth, and resilience to viral escape mutations. Immunity. 2021 Aug 10;54(8):1853–1868.e7. doi:10.1016/j.immuni.2021.07.008. 34331873 PMC8323339

[57] Ubah OC, Lake EW, Gunaratne GS, et al. Mechanisms of SARS-CoV-2 neutralization by shark variable new antigen receptors elucidated through X-ray crystallography. Nat Commun. 2021 Dec 16;12(1):7325. doi:10.1038/s41467-021-27611-y. 34916516 PMC8677774

[58] Benton DJ, Wrobel AG, Xu P, et al. Receptor binding and priming of the spike protein of SARS-CoV-2 for membrane fusion. Nature. 2020 Dec;588(7837):327–330. doi:10.1038/s41586-020-2772-0. 32942285 PMC7116727

[59] Li W, Chen Y, Prévost J, et al. Structural basis and mode of action for two broadly neutralizing antibodies against SARS-CoV-2 emerging variants of concern. Cell Rep. 2022 Jan 11;38(2):110210. doi:10.1016/j.celrep.2021.110210. 34971573 PMC8673750

[60] Wang L, Zhou T, Zhang Y, et al. Ultrapotent antibodies against diverse and highly transmissible SARS-CoV-2 variants. Science (New York, NY). 2021 Aug 13;373(6556), doi:10.1126/science.abh1766. PMC9269068 34210892

[61] Du S, Liu P, Zhang Z, et al. Structures of SARS-CoV-2 B.1.351 neutralizing antibodies provide insights into cocktail design against concerning variants. Cell Res. 2021 Oct;31(10):1130–1133. doi:10.1038/s41422-021-00555-0. 34433900 PMC8385480

[62] Li T, Han X, Gu C, et al. Potent SARS-CoV-2 neutralizing antibodies with protective efficacy against newly emerged mutational variants. Nat Commun. 2021 Nov 2;12(1):6304. doi:10.1038/s41467-021-26539-7. 34728625 PMC8563728

[63] Dejnirattisai W, Zhou D, Ginn HM, et al. The antigenic anatomy of SARS-CoV-2 receptor binding domain. Cell. 2021 Apr 15;184(8):2183–2200.e22. doi:10.1016/j.cell.2021.02.032. 33756110 PMC7891125

[64] Robbiani DF, Gaebler C, Muecksch F, et al. Convergent antibody responses to SARS-CoV-2 in convalescent individuals. Nature. 2020 Aug;584(7821):437–442. doi:10.1038/s41586-020-2456-9. 32555388 PMC7442695

[65] Dong J, Zost SJ, Greaney AJ, et al. Genetic and structural basis for SARS-CoV-2 variant neutralization by a two-antibody cocktail. Nat Microbiol. 2021 Oct;6(10):1233–1244. doi:10.1038/s41564-021-00972-2. 34548634 PMC8543371

[66] Ge J, Wang R, Ju B, et al. Antibody neutralization of SARS-CoV-2 through ACE2 receptor mimicry. Nat Commun. 2021 Jan 11;12(1):250. doi:10.1038/s41467-020-20501-9. 33431856 PMC7801515

[67] Yuan M, Wu NC, Zhu X, et al. A highly conserved cryptic epitope in the receptor binding domains of SARS-CoV-2 and SARS-CoV. Science (New York, NY). 2020 May 8;368(6491):630–633. doi:10.1126/science.abb7269. PMC7164391 32245784

[68] Zhou D, Duyvesteyn HME, Chen CP, et al. Structural basis for the neutralization of SARS-CoV-2 by an antibody from a convalescent patient. Nat Struct Mol Biol. 2020 Oct;27(10):950–958. doi:10.1038/s41594-020-0480-y. 32737466

[69] Asarnow D, Wang B, Lee WH, et al. Structural insight into SARS-CoV-2 neutralizing antibodies and modulation of syncytia. Cell. 2021 Jun 10;184(12):3192–3204.e16. doi:10.1016/j.cell.2021.04.033. 33974910 PMC8064868

[70] Tortorici MA, Czudnochowski N, Starr TN, et al. Broad sarbecovirus neutralization by a human monoclonal antibody. Nature. 2021 Sep;597(7874):103–108. doi:10.1038/s41586-021-03817-4. 34280951 PMC9341430

[71] Liu C, Zhou D, Nutalai R, et al. The antibody response to SARS-CoV-2 beta underscores the antigenic distance to other variants. Cell Host Microbe. 2022 Jan 12;30(1):53–68.e12. doi:10.1016/j.chom.2021.11.013. 34921776 PMC8626228

[72] Sun D, Sang Z, Kim YJ, et al. Potent neutralizing nanobodies resist convergent circulating variants of SARS-CoV-2 by targeting diverse and conserved epitopes. Nat Commun. 2021 Aug 3;12(1):4676. doi:10.1038/s41467-021-24963-3. 34344900 PMC8333356

[73] Koenig PA, Das H, Liu H, et al. Structure-guided multivalent nanobodies block SARS-CoV-2 infection and suppress mutational escape. Science (New York, NY). 2021 Feb 12;371(6530), doi:10.1126/science.abe6230. PMC7932109 33436526

[74] Walls AC, Xiong X, Park YJ, et al. Unexpected receptor functional mimicry elucidates activation of coronavirus fusion. Cell. 2019 Feb 21;176(5):1026–1039.e15. doi:10.1016/j.cell.2018.12.028. 30712865 PMC6751136

[75] Jackson CB, Farzan M, Chen B, et al. Mechanisms of SARS-CoV-2 entry into cells. Nat Rev Mol Cell Biol. 2022 Jan;23(1):3–20. doi:10.1038/s41580-021-00418-x. 34611326 PMC8491763

[76] Pinto D, Sauer MM, Czudnochowski N, et al. Broad betacoronavirus neutralization by a stem helix-specific human antibody. Science (New York, NY). 2021 Sep 3;373(6559):1109–1116. doi:10.1126/science.abj3321. PMC9268357 34344823

[77] Zhou P, Yuan M, Song G, et al. A human antibody reveals a conserved site on beta-coronavirus spike proteins and confers protection against SARS-CoV-2 infection. Sci Transl Med. 2022 Mar 23;14(637):eabi9215. doi:10.1126/scitranslmed.abi9215. 35133175 PMC8939767

[78] Hsieh CL, Werner AP, Leist SR, et al. Stabilized coronavirus spike stem elicits a broadly protective antibody. Cell Rep. 2021 Nov 2;37(5):109929. doi:10.1016/j.celrep.2021.109929. 34710354 PMC8519809

[79] Sauer MM, Tortorici MA, Park YJ, et al. Structural basis for broad coronavirus neutralization. Nat Struct Mol Biol. 2021 Jun;28(6):478–486. doi:10.1038/s41594-021-00596-4. 33981021

[80] Wang C, van Haperen R, Gutiérrez-Álvarez J, et al. A conserved immunogenic and vulnerable site on the coronavirus spike protein delineated by cross-reactive monoclonal antibodies. Nat Commun. 2021 Mar 17;12(1):1715. doi:10.1038/s41467-021-21968-w. 33731724 PMC7969777

[81] Suryadevara N, Shrihari S, Gilchuk P, et al. Neutralizing and protective human monoclonal antibodies recognizing the N-terminal domain of the SARS-CoV-2 spike protein. Cell. 2021 Apr 29;184(9):2316–2331.e15. doi:10.1016/j.cell.2021.03.029. 33773105 PMC7962591

[82] Wang Z, Muecksch F, Cho A, et al. Analysis of memory B cells identifies conserved neutralizing epitopes on the N-terminal domain of variant SARS-Cov-2 spike proteins. Immunity. 2022 Apr 7, doi:10.1016/j.immuni.2022.04.003. PMC8986478 35447092

[83] Yu Y, Wang M, Zhang X, et al. Antibody-dependent cellular cytotoxicity response to SARS-CoV-2 in COVID-19 patients. Signal Transduct Target Ther. 2021 Sep 24;6(1):346. doi:10.1038/s41392-021-00759-1. 34561414 PMC8463587

[84] Adeniji OS, Giron LB, Purwar M, et al. COVID-19 Severity Is associated with differential antibody Fc-mediated innate immune functions. mBio. 2021 Apr 20;12(2), doi:10.1128/mBio.00281-21. PMC8092230 33879594

[85] Wang K, Jia Z, Bao L, et al. Memory B cell repertoire from triple vaccinees against diverse SARS-CoV-2 variants. Nature. 2022 Mar;603(7903):919–925. doi:10.1038/s41586-022-04466-x. 35090164 PMC8967717

[86] Morens DM, Taubenberger JK, Fauci AS. Universal coronavirus vaccines - An urgent need. N Engl J Med. 2022 Jan 27;386(4):297–299. doi:10.1056/NEJMp2118468. 34910863 PMC11000439

[87] He WT, Musharrafieh R, Song G, et al. Targeted isolation of diverse human protective broadly neutralizing antibodies against SARS-like viruses. Nat Immunol. 2022 Jun;23(6):960–970. doi:10.1038/s41590-022-01222-1. 35654851 PMC10083051

[88] Burnett DL, Jackson KJL, Langley DB, et al. Immunizations with diverse sarbecovirus receptor-binding domains elicit SARS-CoV-2 neutralizing antibodies against a conserved site of vulnerability. Immunity. 2021 Dec 14;54(12):2908–2921.e6. doi:10.1016/j.immuni.2021.10.019. 34788600 PMC8554075

[89] Cao Y, Hao X, Wang X, et al. Humoral immunogenicity and reactogenicity of CoronaVac or ZF2001 booster after two doses of inactivated vaccine. Cell Res. 2022 Jan;32(1):107–109. doi:10.1038/s41422-021-00596-5. 34862467 PMC8640508

[90] Tian S, Ji K, Wang M, et al. Distinct BCR repertoires elicited by SARS-CoV-2 RBD and S vaccinations in mice. Cell Discov. 2021 Oct 7;7(1):91. doi:10.1038/s41421-021-00331-9. 34620836 PMC8495183

